# A Sequence Distance Graph framework for genome assembly and analysis

**DOI:** 10.12688/f1000research.20233.1

**Published:** 2019-08-23

**Authors:** Luis Yanes, Gonzalo Garcia Accinelli, Jonathan Wright, Ben J. Ward, Bernardo J. Clavijo

**Affiliations:** 1 Earlham Institute, Norwich, Norfolk, NR4 7UZ, UK

**Keywords:** Genome graph, genome assembly

## Abstract

The Sequence Distance Graph (SDG) framework works with genome assembly graphs and raw data from paired, linked and long reads. It includes a simple deBruijn graph module, and can import graphs using the graphical fragment assembly (GFA) format. It also maps raw reads onto graphs, and provides a Python application programming interface (API) to navigate the graph, access the mapped and raw data and perform interactive or scripted analyses. Its complete workspace can be dumped to and loaded from disk, decoupling mapping from analysis and supporting multi-stage pipelines. We present the design and

implementation of the framework, and example analyses scaffolding a short read graph with long reads, and navigating paths in a heterozygous graph for a simulated parent-offspring trio dataset.

SDG  is  freely  available  under  the  MIT  license  at https://github.com/bioinfologics/sdg

## Introduction

Sequence graphs are the core representation of genome assemblers ^1– 3^ Their use has increased lately thanks to the graphical fragment assembly (GFA) format for graph exchange ^4^, tools to work with genome variation graphs ^5^, and sequence to graph mappers ^6– 10^ But a lack of inter operation between graph-based tools, and limited tools for downstream graph-based analysis, contribute to a perceived complexity which maintains linear sequences as the typical unit of exchange. This flattening of graph representations within pipelines with multiple steps, that use different types of sequencing in an iterative fashion, produces ever-longer linear genome sequences through an information loss process. As a result, genome assembly projects are prone to error propagation and difficult to reproduce and control. These problems can be addressed developing graph-based frameworks to integrate the analysis of hybrid datasets.

The Sequence Distance Graph (SDG) framework implements a **SequenceDistanceGraph** representation that defines sequences in nodes and their adjacency in links, and an associated **Workspace** containing raw data and mappings. This provides an integrated working environment to use multiple sources of information to navigate and analyse genome graphs. **Datastores** allow random access to short, linked, and long read sequences on disk. A mapper on each datastore contains methods to map the reads to the graph and access the mapping data. **KmerCounters** provide functions to compute *k-mer* coverage over the graph from sequencing data, enabling coverage analyses. Additional **DistanceGraphs**, typically representing longer-range information and different linkage levels, define alternative topologies over the **SequenceDistanceGraph** nodes. Finally, a **NodeView** abstraction provides a proxy to a node, with methods to navigate the graph and access its mapped data. This comprehensive framework can be used to explore genome graphs interactively or to create processing methods for assembly or downstream analysis.

Here we describe the SDG implementation and basic tools, providing examples of use cases that highlight its analytic flexibility. First, we show how to create a hybrid assembly by integration of long reads linkage into a short-read graph. Then we analyse a simulated parent-child trio and show how the coverage of the parent datasets can be used to navigate the graph topology. These are only two of the multiple ways integrating data and genome graphs can be used to perform simple but powerful analyses.

## Methods

### Implementation

The C++ core library implements SDG’s data structures and methods for **WorkSpaces**, graphs, datastores and mappers. Its main goal is to provide a straightforward interface to project information from raw datasets onto graphs, and enable easy access and analysis of the graph-data combination. It uses OpenMP for parallel processing, and SWIG 4.0 to export a Python API to enable interactive data analysis.

**Figure 1.**
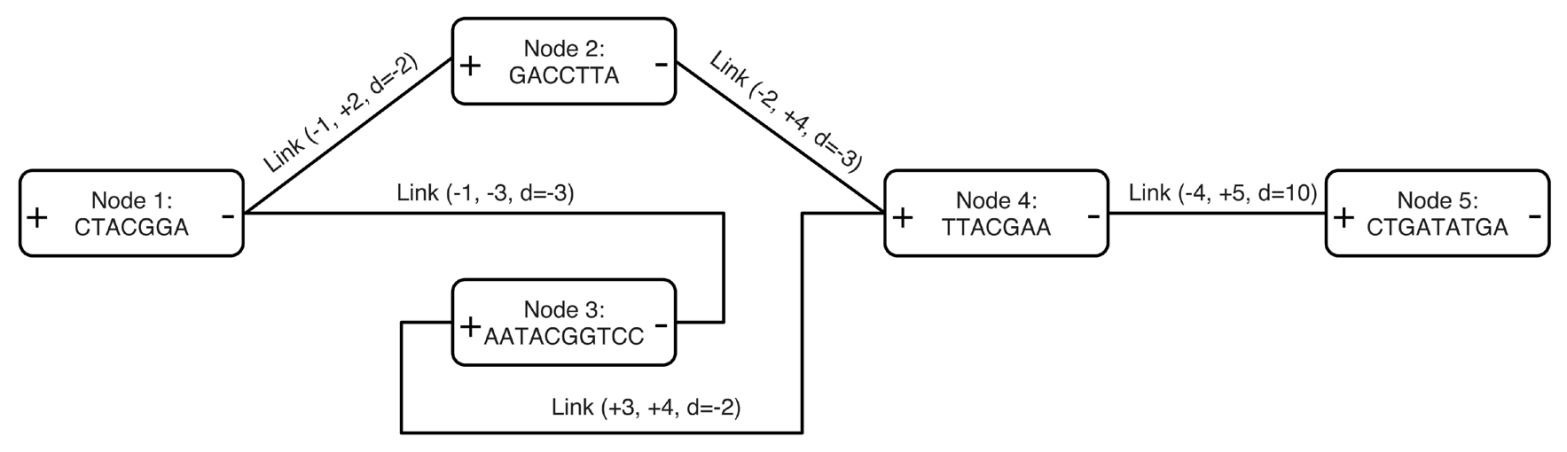
A simple Sequence Distance Graph with 5 nodes, including links with d<0, representing overlaps, and a link representing a gap of 10bp. Sequences appear in only one direction and their reverse complement can be obtained by traversing the node in opposite direction, from - to +. The two largest possible paths are [1, 2, 4, 5] and [1, -3, 4, 5], and their reverse complements [-5, -4, -2, -1] and [-5, -4, 3, -1] respectively.

The **SequenceDistanceGraph** class contains a vector of nodes defining DNA sequences, and a vector of links. Every node has a positive and a negative end, and links are defined between these node ends. Links with positive distances represent gaps between linked sequences and negative distances represent overlaps. This representation, shown in Figure 1, is similar to those presented in 2, 11 but unifies the concept of overlap and gap. Paths can be defined as list of nodes, with the sign of the first end in the walk. Graphs can be read and written to GFA and GFA2 files.

The **DistanceGraph** class contains a set of links over the nodes of a **SequenceDistanceGraph** object. It is used to represent alternative sources of linkage information, such as longer range linkage produced by mapped reads for scaffolding.

The **WorkSpace** contains a single **SequenceDistanceGraph**, multiple **DistanceGraphs**, datastores and mappers, and its structure in memory represents the status of the SDG framework. It can be dumped and loaded from disk, providing persistence and checkpoints between different steps on SDG-based pipelines. Raw reads and *k-mer* counts are kept in separate files, pointed from the **WorkSpace**, to avoid duplication when using multiple **WorkSpaces** around the same dataset.

The **DataStores** and **Mappers** provide access and management to raw data and its mapping on the graph. **Datastores** do not load read data into memory, but rather provide random access to the on-disk data. The **PairedRedMapper** and **LinkedReadMapper** classes use a unique *k-mer* index to map reads to single nodes, with single reads mapping to multiple nodes not being mapped ^3, 12^ The **LongReadMapper** class generates multiple mappings from each read to nodes, using a short non-unique *k-mer* index (k=15 by default) ^13, 14^ Long read mapping filtering is left to later stages of the processing.

The **KmerCounters** creates an index with all the *k-mers* at a given k up to k=31 and counts occurrences of these *k-mers* on the graph, allowing then to count occurrences in datastores or fastq files. These counts, persisted in the **KmerCounter** with a name, can be then accessed to perform *k-mer* coverage analyses. Projections of raw *k-mer* coverage in the reads and the assembly over a particular sequence for a node or path, similar to those produce by the "sect" tool of K-mer Analysis Toolkit (KAT) ^15^ are valuable for content analysis. Spectra analysis of these frequencies can provide further insight into genome composition and representation on the assembly.

Two processing classes, **LinkageUntangler** and **LinkageMaker**, work with alternative linkage configurations. The **LinkageMaker** is used to condense information via one of its make_linkage* methods, from evidence in the **WorkSpace** into links in a **DistanceGraph**. The **LinkageUntangler** class works on a **DistanceGraph** to simplify, condense and/or linearise its linkage. In the second use case below it can be seen how a combination of **LinkageMaker** and **LinkageUntangler** can be used for scaffolding with long reads.

Finally, the **NodeView** class, and its associated **LinkViews**, provide a single-entry point for node-centric analyses. A **NodeView** from either a **DistanceGraph** or **SequenceDistanceGraph** is a wrapper containing a pointer to the graph and a node id, and will provide access to its nodes’ previous and next linked nodes, mapped reads, or *k-mer* coverage. A user with good understanding of the **NodeView** class should be able to access most information in the **WorkSpace** through it, making it the default choice for analysing the graph.

### Operation

***Requirements and installation.*** SDG can be run on Linux and MacOS, and requires enough RAM to hold the WorkSpace completely in memory, which will depend on the dataset. Space to hold the uncompressed sequences on the datastores on disk will also be required.

SDG can be installed via pre-compiled binaries from https://github.com/bioinfologics/sdg/releases. The binaries have been built using Python3 and GCC version 6 from the Ubuntu package manager for the Linux version. The MacOS version dependencies were obtained using Homebrew (Python3, GCC-6 and SWIG). SDG can be compiled using CMake, Python3, SWIG version 4 and GCC version 6 onwards. Detailed instructions can be found at https://bioinfologics.github.io/sdg/sdg/README.html#installation.

***Typical workflow.*** Working with SDG typically involves two different stages: creating a **WorkSpace** with the data and mappings, and analysing this **WorkSpace**. SDG includes command line tools to create **DataStores**, **KmerCounts**, and **WorkSpaces**, and map reads within a **WorkSpace**.

- **sdg-datastore**: creates a **Datastore** from raw reads and can process paired, 10x or long reads. An output prefix is specified as a parameter and a <prefix>.prseq, <prefix>.lrseq or <prefix>.loseq file is generated.
- **sdg-kmercounter**: creates a **KmerCounter** indexing a graph from a **WorkSpace** or GFA, or works with an already generated one. A count can be added directly from raw reads or from a datastore. The **KmerCounter** is persisted on file with extension ’sdgkc’.
- **sdg-workspace**: creates a **WorkSpace** from a base graph or works with an already generated one. **Datastores** and **KmerCounters** can be added. The **WorkSpace** is persisted on file with extension ’sdgws’.
- **sdg-dbg**: creates a **WorkSpace** from a **PairedReadDatastore** by building a *deBruijn graph* and using this as the base graph. Counts for the *k-mers* from the graph and raw reads are added too.
- **sdg-mapper**: maps reads within a **WorkSpace**. An updated **WorkSpace** is produced and dumped to the specified prefix.

**WorkSpaces** can also be instantiated with an empty graph, and the graph populated through the add_node and add_link methods. The following example on a python session shows how the simple graph from Figure 1 can be created from scratch, navigated through a **NodeView** instance and sequence from its paths extracted.


>>> import pysdg as SDG
version 0.1
master b4d3f02
>>> ws=SDG.WorkSpace()
>>> ws.sdg.add_node("CTACGGA")
1
>>> ws.sdg.add_node("GACCTTA")
2
>>> ws.sdg.add_node("AATACGGTCC")
3
>>> ws.sdg.add_node("TTACGAA")
4
>>> ws.sdg.add_node("CTGATATGA")
5
>>> ws.sdg.add_link(-1, 2, -2)
>>> ws.sdg.add_link(-1, -3, -3)
>>> ws.sdg.add_link(-2, 4, -3)
>>> ws.sdg.add_link(3, 4, -2)
>>> ws.sdg.add_link(-4, 5,
                         10)
>>> nv=ws.sdg.get_nodeview(1)
>>> nv
<NodeView: Node 1 in SDG>
>>> nv.next()
<Vector: 2 LinkViews>
>>> print(nv.next())
[
<LinkView: -3bp to Node -3>,
<LinkView: -2bp to Node 2>
]
>>> nv = nv.next()[0].node()
>>> nv
<NodeView: Node -3 in SDG>
>>> print(nv.prev())
[
<LinkView: -3bp to Node 1>
]
>>> nv.sequence()
'GGACCGTATT'
>>> SDG.SequenceDistanceGraphPath(ws.sdg, [1, -3, 4, 5]).sequence()
'CTACGGACCGTATTACGAANNNNNNNNNNCTGATATGA'


**Figure 2.**
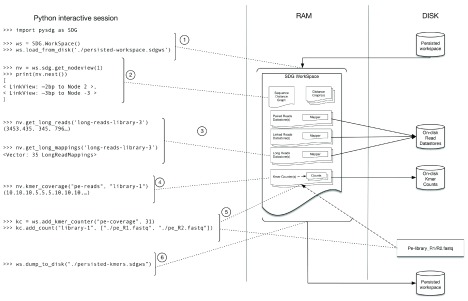
Structure of a WorkSpace and access via an Python interactive session. The WorkSpace holds the information for a project and contains the graphs, the mappers and *k-mer* counts. From Python, a previously saved WorkSpace is loaded from disk (1). The NodeView object is centred on a specific node and can be used to access node characteristics (ie. size and sequence), graph topology from the perspective of the node you are on (i.e. neighbours in both directions (2)) and can also retrieve information projected onto the selected node (ie. mappings (3) and *k-mer* coverage (4)). Operations such as adding a KmerCounter to the WorkSpace and adding a count (5) can be performed, and the WorkSpace can be saved back to disk (6). Once loaded, the bulk of the WorkSpace is held in memory for fast access with the raw read data from the DataStores remaining on disk accessible through random access.

Typically, as shown in Figure 2, the API is used to explore a larger **WorkSpace**, with the methods accessing both in-memory and on-disk data, and modifying the status of the **WorkSpace**.

## Example use cases

To illustrate the use of SDG, we have reproduced a short version of two examples from http://bioinfologics. github.io/sdg_examples.

All paired end datasets are available on https://zenodo.org/record/3363871#.XUwyVy2ZN24 ^16^, and the PacBio reads are from NCBI accession PRJNA194437 ^17^ For simplicity, we have also made the datasets available on https://opendata.earlham.ac.uk/opendata/data/sdg_datasets/ as ready-to-use ’fastq.gz’ files.

### Hybrid assembly of short and long reads

This example is based on an *E. coli* dataset combining PacBio reads from 17 and Illumina Miseq 2x300bp reads subsampled from a test run. It uses the long reads to scaffold a short read based graph produced by *sdg-dbg*. Graphs are dumped to GFA files at different stages, and visualised using Bandage v0.8.1 ^18^

First, we use the command line tools to create datastores for both long and short reads and an initial **WorkSpace** containing a DBG assembly:


sdg-datastore make -t paired -o ecoli_pe ../ecoli_pe_r1.fastq.gz -2 ../ecoli_pe_r2.fastq.gzsdg-datastore make -t long -o ecoli_pb -L ../ecoli_pb_all.fastq.gz
sdg-dbg -p ecoli_pe.prseq -o ecoli_assm


From this point on, we use the python SDG library. First, we load the workspace, add a long read datastore and map its reads using a k=11 index.


importpysdgasSDG
# Load sdg-dbg's workspace from disk, add the pacbio datastore
ws=SDG.WorkSpace('ecoli_assm.sdgws')
lords=ws.add_long_reads_datastore('ecoli_pb.loseq')
# Map long reads
lords.mapper.k= 11
lords.mapper.map_reads()

ws.sdg.write_to_gfa1('initial_graph.gfa')


**Figure 3.**
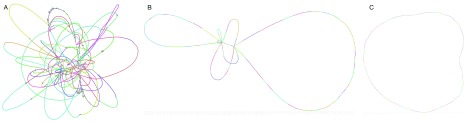
Linkage at different stages of the long read scaffolding example, visualised using Bandage: **A**) SequenceDistanceGraph generated by sdg-dbg from short reads, **B**) DistanceGraph generated after using make_nextselected_linkage on the long read data, linking all nodes of 1100bp and more, **C**) DistanceGraph after eliminating all nodes with multiple connections (repeats).

The graph, as shown in Figure 3A contains multiple unresolved repeats.

We can use the LinkageMaker to create linkage using the long reads datastore. We do this by selecting the nodes between which to analyse possible linkage, in this case all nodes of 1100bp or more, and then calling the make_longreads_multilinkage method, with alignment filtering parameters of 1000bp and 10% id.


lm=SDG.LinkageMaker(ws.sdg)
lm.select_by_size(1100)
mldg=lm.make_longreads_multilinkage(ws.long_reads_datastores[0].mapper, 1000, 
                        10)


This multi-linkage can be collapsed using the LinkageUntangler. The make_nextselected_linkage method links every selected node to its closest selected neighbours on each direction, aggregating the distances via a simple median calculation:


lu=SDG.LinkageUntangler(mldg)
lu.select_by_size(1100)
ns_dg=lu.make_nextselected_linkage()
ns_dg.write_to_gfa1('ns_collapsed.gfa')


The new graph we dumped, as shown in Figure 3B, has disconnected the repeats and introduced long read linkage which skips over them, but it is still not fully solved. We can improve this further by getting rid of repetitive nodes that will be connected to multiple neighbours, as each of them belongs in more than one place. We do that by just turning these nodes’ selection off in the **LinkageUntangler**, which will then skip them in the solution.


fornvinns_dg.get_all_nodeviews():
if len(nv.prev())> 1orlen(nv.next())> 1:
lu.selected_nodes[nv.node_id()]=False
ns_nr_dg=lu.make_nextselected_linkage()

ns_nr_dg.write_to_gfa1('ns_nr_final.gfa')


The last graph is now a circle, with all the repeats disconnected from any linkage.

### Analysing a simulation of heterozygous parent-child trio with short reads

We created a simulation of a trio dataset for this example using the synthetic genome creation and sequencing package Pseudoseq.jl v0.1.0 ^19^ Chromosomes 4 and 5 of the reference genome of the yeast strain S288C were used as templates to create a diploid, genome for each parent with 1% heterozygous sites. Each homologous pair of chromosomes was crossed over and recombined and the child inherited one chromosome from the first parent at random, and one chromosome from the second parent at random. Simulated paired end reads were generated for each genome, using an average fragment length of 700bp and a read length of 250bp, and an expected coverage of 70x with error rate was set to 0.1%.

First we used the command line tools to create a graph from the child reads using sdg-dbg, and add *k-mer* counts for both parents into the datastore.


sdg-datastore make -t paired -1 child/child-pe-reads_R1.fastq.gz -2 child/child-pe-reads_R2.fastq.gz -o child_pe
sdg-dbg sdg-dbg -p child_pe.prseq -o sdg_child
sdg-kmercounter add -c main.sdgkc -n p1 -f p1/p1-pe-reads_R1.fastq.gz -f p1/p1-pe-reads_R2.fastq.gz -o main
sdg-kmercounter add -c main.sdgkc -n p2 -f p2/p2-pe-reads_R1.fastq.gz -f p2/p2-pe-reads_R2.fastq.gz -o main


We now open the **WorkSpace** and use the **NodeView::parallels** method to look for the largest bubble structure in the graph, which should be formed by two parallel nodes with haplotypes coming from each parent.


import pysdgas SDG
ws = SDG.WorkSpace('sdg_child.sdgws')
#Largest node with one parallel node, and its parallel
maxbubble = 0
for nv in ws.sdg.get_all_nodeviews():
if nv.size() > maxbubble andlen(nv.parallels()) == 1:
maxbubble=nv.size()
bubble_nvs=(nv,nv.parallels()[0])


Since each side should be a haplotype from a different parent, we should see a loss of *k-mer* coverage on the parent that didn’t contribute that haplotype. To check this, we create a plotting function to plot the output from the **NodeView::kmer_coverage** method.


defplot_kcov(nv):
'''Plot kmer coverage across the three read sets. Requires pylab.'''
figure();suptitle("Coverage for "+str(nv));
subplot(3,1,1);ylim((0,120))
plot(nv.kmer_coverage("main","PE"), label="child"); legend(loc=1);
subplot(3,1,2);ylim((0,120))
plot(nv.kmer_coverage("main","p1"),"red", label="parent 1"); legend(loc=1);
subplot(3,1,3);ylim((0,120))
plot(nv.kmer_coverage("main","p2"),"blue", label="parent 2"); legend(loc=1);

plot_kcov(bubble_nvs[0])
plot_kcov(bubble_nvs[1])


**Figure 4.**
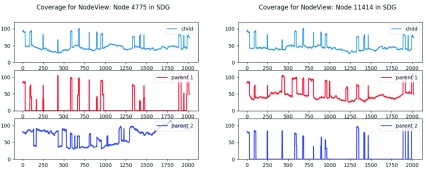
Trio analysis: *k-mer* coverage for each side of the largest bubble structure in the child’s assembly by each of the three read sets. Coverage drops to 0 on the opposite parent for *k-mers* that are unique to a parent.


The plots, shown in Figure 4, reflect how Node 4775 contains content inherited from parent 2 and its parallel node 11414 contains content inherited from parent 1. We can create a function to extend these parent-specific regions by walking forward and backward as long as only one link takes us to a node that is fully covered by the content of the parent we are following.

defextend_parent_covered_path(starting_node,target_parent):
ifws.sdg.get_nodeview(starting_node).kmer_coverage("main", 
                        target_parent).count(0)!= 0:
returnSDG.SequenceDistanceGraphPath(ws.sdg,[])
p=SDG.SequenceDistanceGraphPath(ws.sdg,[starting_node])
forxin[0,
                        1]:
nv=ws.sdg.get_nodeview(p.nodes[-1])
whilenv.next():
next_node= 0
fornlinnv.next():
ifnl.node().kmer_coverage("main", 
                        target_parent).count(0)== 0:
ifnext_nodeornl.node().node_id()inp.nodes:
next_node= 0
break
else:
next_node=nl.node().node_id()
ifnext_node== 0:break
p.nodes.append(next_node)
nv=ws.sdg.get_nodeview(next_node)
p.reverse()
returnp
path1=extend_parent_covered_path(11414, 
                        "p1")
path2=extend_parent_covered_path(4775, 
                        "p2")


After using this function, path1 contains 49 nodes yielding 8672bp of sequence inherited from parent 1, and path2 contains 139 nodes yielding 26351bp of sequence inherited from parent 2. It is important to note that the difference in node count and sequence length arises because the extension function is haplotype-specific and its results depend in the topology of each haplotype graph.

## Summary

The Sequence Distance Graph framework provides a unified workspace for different sequencing technologies using the genome graph as the basis of integration. It enables analyses across the graph topology, the raw data and its projections to the graph. We have shown how the NodeView class can be used through the Python API to produce interactive analyses that are both powerful and easy to follow. We expect this will be a useful codebase for all levels of users, not only for the construction of graph-based analysis but also for their teaching and dissemination.

## Data availability

### Source data

The PacBio, *E. coli* reads are deposited on NCBI accession PRJNA194437 from Koren *et al.* ^17^

*E. coli* K12 Re-sequencing with PacBio RS and 454: Accession number PRJNA194437, https://identifiers.org/ncbi/bioproject:PRJNA194437

### Underlying data

The datasets used in the examples are available from: https://opendata.earlham.ac.uk/opendata/data/sdg_datasets/ and archived in Zenodo Zenodo: SDG Paper Datasets. http://doi.org/10.5281/zenodo.3363871 ^16^

Data are available under the terms of the Creative Commons Zero "No rights reserved" data waiver (CC0 1.0 Public domain dedication).

## Software availability

Software documentation: https://bioinfologics.github.io/sdg

Source code available from: http://github.com/bioinfologics/sdg

Archieved source code at time of publication: https://zenodo.org/record/3363165#.XUw1yy2ZN25 ^20^

License: MIT License
