# Supplementary material for: A Sequence Distance Graph framework for genome assembly and analysis
Source: F1000Res. 2019 Aug 23;8:1490. [Version 1] doi: 10.12688/f1000research.20233.1 (PMC6833988; doi:10.12688/f1000research.20233.1)
Supplement: Peer review history

# Peer review: Reviewer response for version 1

**DOI:** 10.5256/f1000research.22229.r55755

**Published:** 2020-01-24

**Authors:** Max Alekseyev, Pavel Avdeyev

**Affiliations:** 1 George Washington University, Washington, DC, USA

The authors describe a software package aimed at construction, storage, and manipulation of sequence graphs. The software is publicly available on Github. While the core functionality is developed in C++, the package also provides Python wrapper library and a command-line tool. The authors outline the software design and provide pipelines and code examples for two different types of data.

    Overall, the paper is well-written and describes potentially useful software. At the same time, the paper lacks:

1. Comparison of (features of) the developed software and existing software such as VG toolkit based on variation graphs (reference 5 ^1^);
2. Discussion of the software applicability (e.g., will it work on large or repeat-rich genomes?), or estimation of its running time/space complexity.

Some minor comments:

1. The use of bold font is not explained. For example, Datastores first appears in the sentence “ **Datastores** allow random access...” describing its features, but WHAT is Datastores?
2. Orthogonal edge routing in Fig. 1 is somewhat confusing, why not make edges curved?

We confirm that we have read this submission and believe that we have an appropriate level of expertise to confirm that it is of an acceptable scientific standard, however we have significant reservations, as outlined above.

## References

[1] : Variation graph toolkit improves read mapping by representing genetic variation in the reference. *Nature Biotechnology*.2018;36(9) : 10.1038/nbt.4227 875-879 10.1038/nbt.4227 PMC612694930125266

# Peer review: Reviewer response for version 1

**DOI:** 10.5256/f1000research.22229.r55323

**Published:** 2019-11-04

**Authors:** Eric T. Dawson

**Affiliations:** 1 Division of Cancer Epidemiology and Genetics, National Cancer Institute, Rockville, MD, USA; 2 Department of Genetics, University of Cambridge, Cambrige, UK

The authors describe a framework for constructing sequence graphs, aligning reads, manipulating graph structures, and extracting them into standard formats (e.g., GFA). This framework is available as both a set of command line tools and a python library which wraps much of the underlying functionality. Their implementation unifies the representation of gaps and overlaps as a single linkage type within the graph. This is the primary theoretical advance of the work. This work is scientifically sound but its description as written could benefit from some minor additions.

 The software is freely available on GitHub and binary releases of the command line tools are provided. These are functional on a modern linux laptop and clear examples with data are provided. The paper includes the outputs of these examples as figures.

 The python libraries rely on SWIG and are not included in the binaries. While not requisite for publication, providing the python libraries through pip, conda, or another package manager would increase the reach of the framework. This would match the authors' conclusion that the sdg package provides "a useful codebase for all levels of users."

 The examples provided are clear and scientifically relevant. The graph mapping and manipulation example using *E. coli *data (Figure 3) and the description of genotyping a simulated yeast trio (Figure 4) are both realistic.

 However, the authors should provide run times and machine details for these examples. Both are relatively fast as the datasets are small. There is no need for extensive benchmarking; a footnote for each example would address this adequately.

 A brief 1-2 sentence discussion of a larger scale example the authors have attempted should also be included.

 In addition, the phrasing "simulated parent-offspring trio" in the abstract should be modified to make it clear that the data is from yeast. As it is written the phrasing implies the framework may work on human/animal-scale data, though no evidence of this has been provided in this version of the paper.

 Lastly, a brief description of the similarities and differences between the sequence (distance) graph, the variation graph, and the de Bruijn graph from an assembler such as ABySS should be included in the introduction or provided by a reference. This description need not be longer than two to four sentences in length. This should highlight the similar representations of the graphs (e.g., sequences stored in nodes and linkages/paths described by edges) and the different amounts of information content within the graph types. This would strengthen the critical need for the software and is partially highlighted by the example in Figure 3.

 As it stands the paper is deserving of indexing. These additions would further strengthen what is already an excellent tool description, I hope without adding too much additional work for the authors.

I confirm that I have read this submission and believe that I have an appropriate level of expertise to confirm that it is of an acceptable scientific standard.

# Peer review: Reviewer response for version 1

**DOI:** 10.5256/f1000research.22229.r52959

**Published:** 2019-09-24

**Authors:** Erik Garrison

**Affiliations:** 1 University of California, Santa Cruz, Santa Cruz, CA, USA

The authors demonstrate a new toolchain and data model for working with sequence graphs. This method allows the user to dynamically interact with sequence graphs made in the process of assembly. They provide a number of examples of the use of the method as well as code snippets to demonstrate its functionality. The library is written in C++, but wrapped in python with SWIG, which should make it useful to many researchers for whom C++ is difficult to use.

 I find only one thing strange about the work. In the beginning, the authors indicate that there are not interoperable methods for working with sequence graphs and alignments to them, but they have in effect created another competing standard. Are there particular limitations with existing data models that they hope to address with the Sequence Distance Graph framework? How is their model different than the variation graph model, in which distances are provided by a collection of paths (or equivalently alignments) embedded within the sequence graph?

I confirm that I have read this submission and believe that I have an appropriate level of expertise to confirm that it is of an acceptable scientific standard.
